# Therapeutic Options for Childhood Absence Epilepsy

**DOI:** 10.3390/pediatric13040078

**Published:** 2021-12-16

**Authors:** Victoria Elisa Rinaldi, Giuseppe Di Cara, Elisabetta Mencaroni, Alberto Verrotti

**Affiliations:** 1 Pediatric Unit, San Giovanni Battista Hospital, 06034 Foligno, Italy; 2 Pediatric Unit, Department of Medicine and Surgery, University of Perugia, 06156 Perugia, Italy; giuseppe.dicara@unipg.it (G.D.C.); elisabettamencaroni@gmail.com (E.M.); alberto.verrottidipianella@unipg.it (A.V.)

**Keywords:** childhood absence epilepsy, absences, epilepsy treatment, resistant epilepsy

## Abstract

Childhood absence epilepsy (CAE) is a common pediatric generalized epileptic syndrome. Although it is traditionally considered as a benign self-limited condition, the apparent benign nature of this syndrome has been revaluated in recent years. This is mainly due to the increasing evidence that children with CAE can present invalidating neuropsychological comorbidities that will affect them up to adulthood. Moreover, a percentage of affected children can develop drug-resistant forms of CAE. The purpose of this review is to summarize the most recent studies and new concepts concerning CAE treatment, in particular concerning drug-resistant forms of CAE. A Pubmed search was undertaken to identify all articles concerning management and treatment of CAE, including articles written between 1979 and 2021. Traditional anticonvulsant therapy of CAE that is still in use is based on three antiepileptic drugs: ethosuximide which is the drug of choice, followed by valproic acid and lamotrigine. In the case of first line treatment failure, after two monotherapies it is usual to start a bi-therapy. In the case of absence seizures that are refractory to traditional treatment, other antiepileptic drugs may be introduced such as levetiracetam, topiramate and zonisamide.

## 1. Introduction

Childhood absence epilepsy (CAE) is a common pediatric generalized epileptic syndrome that affects between 10% to 17% of all school-aged children diagnosed with new onset epilepsy [1]. It is defined by the presence of multiple daily typical absence seizures that are characterized by a sudden interruption of on-going activities, possibly an upwards rotation of the eyes and a blank stare with impaired consciousness [2]. These episodes of altered awareness can be activated by hyperventilation or photic stimulation and are characterized by generalized symmetrical and synchronous spike-wave discharges at 3 Hz or more on electroencephalogram (EEG) [2], usually lasting between 9 and 10 s [3].

The first descriptions of CAE date back to the last century in German medical literature and in 1916 Sauer presented the term pyknolepsy, from the Greek word piknoz (πικνός), meaning “very frequent or grouped”, to describe absence seizures with daily recurrences [4]. The typical age of onset is between 6 to 7 years but it can also be observed between 4 to 10 years of age in patients with a normal psychomotor development and without a particular personal or family history for neurologic diseases [5].

Recent studies have also shown a possible association of absence seizures and other epileptic syndromes such as idiopathic occipital epilepsy, Gastaut idiopathic occipital epilepsy [6], West syndrome, myoclonic epilepsy of infancy and benign epilepsy with centro-temporal spikes [2,7,8,9].

Most of the molecular-genetic studies on CAE have failed to precisely identify its genetic pathways that could be particularly useful for precision therapy [10]. However, the newest science frontiers are constantly trying to describe the genetic variants associated with this epilepsy, in order to improve disease risk predictability. In particular, most of the genes associated with CAE are ion channel genes (calcium channel, GABA receptor, acetylcholine receptor etc.); in detail, genetic investigations in patients with CAE have demonstrated the role of the calcium channel genes CACNA1H, CACNA1G and CACNG3 along with GABA A and B receptor genes [11,12]. Moreover, absence seizures result principally from disruptions in thalamocortical pathways involving T-type calcium channels and antiepileptic drugs effective against absence seizures among other mechanisms, exert their effect principally at these channels [13]. In fact, recent studies have focused their attention on pretreatment connectivity in children diagnosed with CAE, with the objective of providing clearer insights into antiepileptic drug response variability for these patients [14].

Although CAE is traditionally considered a benign self-limited epilepsy syndrome, data from the multicenter Childhood Absence Epilepsy Study Group have led to the review of this notion [15,16]. During the past years, the apparent benign nature of the syndrome has in fact undergone many contradictions on a few basis, mainly the possibility of treatment failure (5–10% of patients) and the evidence of considerable psychosocial difficulties that can be found up to adulthood [17,18].

In particular, attention deficits are observed in about 1/3 of the patients and there is an increased risk of cognitive disorders [17]. In light of this new knowledge, as already performed for children with other forms of epilepsy [19], it is fundamental for patients with new onset absence epilepsy to undergo neuropsychological assessments and treatment. The reason for an early cognitive dysfunction in CAE remains unclear but cognitive side effects should be taken into consideration in the choice of antiepileptic treatment with a specific assessment during follow up [5].

CAE diagnosis is often straightforward and highly suspected in school-aged children who present with brief multiple daily episodes of staring without memory of the event or ability of caretakers to interrupt the episodes [20]. Episodes can be classically activated by hyperventilation; doctors can evoke this kind of seizure and furthermore, parents can evoke a staring spell even during telemedicine consultations that have become more and more necessary during the past year [20].

Diagnosis is then confirmed by a routine electroencephalography (EEG) before beginning treatment and to diagnose CAE, only typical absences have to be registered. The presence of other types of seizures (tonic-clonic generalized seizures, atypical absences etc.) rules out a diagnosis of CAE. On EEG the typical findings are a bilaterally symmetrical and synchronous discharge of 3-Hz spike wave complexes with an abrupt start and ending [1]; the background EEG track has a normal and well organized rhythm. If the clinical symptoms and the electrical pattern are typical, there is no differential diagnosis from CAE. However, if there is a poor response to classical CAE treatment or in the suspicion of precocious absence epilepsy (before 3–4 years of age), the initial diagnosis must always be revaluated. In fact, especially in younger children, 10% of poor responders have been found to have a deficit in the type GLUT-1 glucose transporter [21]. This diagnosis can be confirmed through a lumber puncture with glycorrhachia measurement and contemporary measurement of venous glycemia, along with genetic studies of the SLC2A1 gene [5].

Traditional prophylactic anticonvulsant therapy of CAE is based on three antiepileptic drugs: ethosuximide (ETX), valproic acid (VPA) and lamotrigine (LTG) [22] and in particular, the 2010 childhood absence epilepsy study provided class I evidence for the use of ETX as the optimal initial treatment of CAE [15,23]. In the case of first line treatment failure, after having reconsidered and confirmed the initial diagnostic suspect, after two monotherapies it is usual to start a bi-therapy (for example the association of VPA and LTG) carefully watching out for adverse effects [5]. In the case of absence seizures that are refractory to traditional treatment, other antiepileptic drugs may be introduced such as levetiracetam, topiramate and zonisamide [24,25,26].

The purpose of this review is to summarize the most recent studies and new concepts concerning CAE treatment, to highlight areas of consensus as well as areas of uncertainty concerning this “not so benign” common childhood epileptic syndrome. In particular, the objective of this paper is to provide an updated overview of the current knowledge concerning CAE treatment. We hope this paper is of easy access to health-care providers dealing with CAE patients. We focalize our attention on the treatment of refractory CAE also underlining that current knowledge concerning these forms is extremely scarce and that further studies are needed in this direction given the “not so benign nature” of this disease. We sincerely hope this paper will be helpful in the development of CAE treatment guidelines.

## 2. Materials and Methods

This article provides an updated overview of the principal therapeutic options for childhood absence epilepsy. A thorough Pubmed search indexed for MEDLINE, Pubmed and EMBASE was undertaken to identify articles concerning management and treatment of CAE, including articles written between 1979 and September 2021. The main keywords used were: childhood absence epilepsy treatment, along with absence seizures, epilepsy treatment, antiepileptic drugs and resistant epilepsy. In particular, a non-systematic identification through Pubmed, MEDLINE, and EMBASE was performed and around 300 papers were initially screened for information relevant to this review. Forty-four of these papers were then selected according to the following inclusion criteria: available full text in either English or French, focus on CAE treatment in the pediatric population and the latest (in the last 10 years) published papers were preferred over previously conducted studies. Original articles, metanalysis, guidelines and systematic reviews along with narrative reviews were preferred over case reports. Publications containing data that were not relevant for the purpose of our review were excluded from our search.

The list of references of all the relevant articles was also studied to include all relevant scientific papers connected with the subject and this led to the inclusion of another 27 articles for a total of 71 articles.

**Figure 1:**
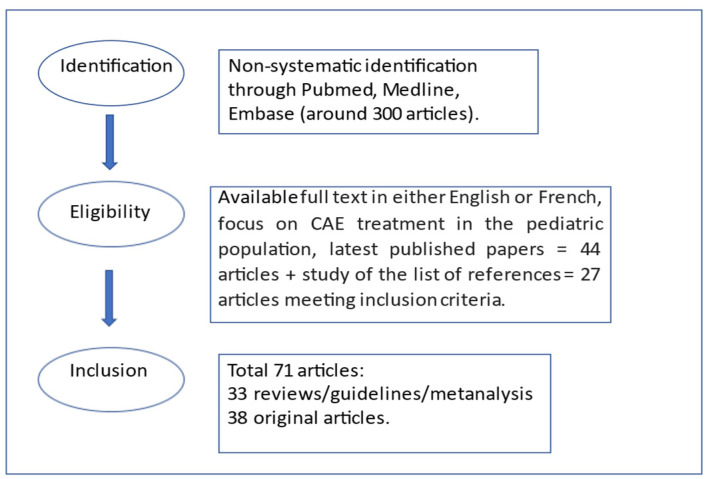
Selection of studies flowchart.

The date of our last search was September 2021. Figure 1 shows the selection of studies flowchart.

## 3. Childhood Absence Epilepsy Therapeutic Options

### 3.1. First Line Treatment

In the last 10 years, various studies have been carried out to compare the different molecules proposed for CAE treatment. However, there are very few randomized trials in literature to guide treatment strategies for CAE. Glauser et al. in 2010 [15] performed a double blind randomized controlled trial comparing the use of ethosuximide, valproate and lamotrigine. A total of 453 children aged between 4 and 10 years with a new diagnosis of CAE were enrolled and were randomly assigned to treatment with ETX, VPA or LTG. The principal outcome that was studied was the freedom from seizures after 16 weeks of treatment and the absence of major adverse effects. The results of this study showed that the freedom from seizures rates of ETX and VPA were similar (53% and 58% respectively, *p* = 0.35) and higher than those of LTG compared to the other two drugs (29%, *p* < 0.001). However, post treatment attention disfunction was more frequent in patients treated with VPA rather than ETX (49% versus 33% respectively, *p* = 0.03) and patients treated with VPA also presented problems related to weight gain in the following months, causing discontinuation of therapy. ETX was therefore considered the drug of choice as initial therapy for CAE [27]. This was also confirmed by Berg et al. in 2014 [28] who demonstrated a higher complete remission rate over 5 years in patients treated with ETX rather than VPA. A recent Cochrane review [29] confirmed these data adding that concerning efficacy and tolerability, ETX represents the optimal initial empirical monotherapy for CAE patients but in the case of coexistence of absences and generalized tonic-clonic seizures, VPA is the drug of choice as ETX has no effect on tonic-clonic seizures. LTG on the other hand, can be preferred if VPA is not considered the drug of choice for a specific patient. To summarize, ethosuximide, lamotrigine and valproate are the principal antiepileptic drugs used to treat children and adolescents with childhood absence epilepsy [30].

#### 3.1.1. Ethosuximide

Ethosuximide (2-ethyl-2-methyl-succinamide) is the drug of choice for classical CAE with simple absence seizures as it does not suppress focal onset or generalized tonic-clonic seizures [23]. Its mechanism of action is not well defined but seems to be based on the blockade of transient, low-threshold calcium currents produced by the thalamus, that cause the synchronous activation of spike wave discharges causing absence seizures [31]. The recommended dosage for CAE is an initial dose of 10–15 mg/kg/day, maintained at 20–30 mg/kg/day divided in two doses (maximum dose 40 mg/kg/day) [23]. Various oral formulations exist (syrup, capsules) and when used in association with other anticonvulsant therapies, it is important to take into consideration its susceptibility to the effects of enzyme inducing and inhibiting antiepileptic drugs [32]. This can happen for example when associating ETX and VPA. The principle side effects of ETX include gastrointestinal disturbances (abdominal discomfort, nausea, vomiting, diarrhea), headache, drowsiness and much rarer side effects such as behavioral and psychiatric disturbances, blood dyscrasias and allergic reactions [23].

#### 3.1.2. Valproic Acid

One of the greatest concerns for pediatric neurologists is to differentiate classic isolated CAE from other forms presenting also with generalized tonic–clonic seizures. This is one of the prevailing reasons to start child treatment with either VPA or LTG as first line treatment, instead of ETX [33]. Valproic acid (N-dipropylacetic acid), is a broad spectrum antiepileptic drug with pre and post-synaptic effects that depend on a very broad spectrum of actions [34], including the regulation of ionic currents and the facilitation of inhibiting GABAergic over glutamatergic transmission. The initial dosage for all oral formulations is 10–15 mg/kg/day, maintained at 20–40 mg/kg/day divided in two doses (maximum dose 60 mg/kg/day) [23]. Contrary to ETX, one of the main problems and causes of discontinuation with VPA therapy are the numerous potential side effects, some of which are dose-related whereas others are idiosynchrasic. In particular, even Glauser et al. in their 2010 trial [15] showed that valproic acid negatively affected patients attention to a greater degree than the other tested drugs. This confirmed that persisting attention problems and neuropsychological dysfunction are an important feature of this syndrome that must be precociously identified when choosing an appropriate antiepileptic treatment [35]. Among the most frequently reported side effects of VPA are increased appetite and weight gain along with the rarer but well known possible occurrence of pancreatitis and hepatic failure [36]. Other potential metabolic side effects include hyperammoniemia, hypothyroidism, hair loss polycistic ovary syndrome and teratogenicity [34]; thrombocytopenia and depletion of coagulation factors can be another dose-dependent side effect of VPA that must be remembered in the case of surgery [23].

#### 3.1.3. Lamotrigine

Lamotrigine acts through blockage of voltage-dependent sodium channels; it also stabilizes presynaptic membranes and inhibits the release of excitatory neurotransmitters, especially glutammate and aspartate [37]. As we have previously underlined, the 2010 double blinded randomized CAE trial [15] has somehow downgraded the use of LTG as first line monotherapy for CAE as the seizure freedom rate after 12 months was lower in patients treated with LTG compared with those treated with ETX or VPA. Moreover, discontinuation for lack of efficacy was more frequently observed in the group treated with LTG. In fact, LTG had previously been considered among first line treatment for CAE and the seizure freedom rates concerning this drug had been reported between 50% and 80% [22,38,39]. However, LTG can be considered as a second monotherapy in the case of ETX failure and VPA is not the appropriate drug for certain patients. For patients not taking other antiepileptic drugs the initial dosage for all oral formulations is 0.6 mg/kg/day, maintained at 5–12 mg/kg/day (maximum dose 300 mg/day) [23]. The initial dosage is very low, and titration must be achieved very slowly over many weeks to avoid Stevens–Johnson Syndrome, a rare but life-threatening adverse effect of this drug. On the other hand, LTG is generally well tolerated and there is a lower risk for cognitive adverse effects compared to VPA although diplopia, dizziness and ataxia have been reported [40].

### 3.2. Associations of Antiepileptic Drugs

The failure of an antiepileptic treatment or any worsening of symptoms during treatment has to alert the pediatric neurologist among other things, to reconsider the etiologic diagnosis of the epileptic syndrome. If the diagnosis of CAE is confirmed, after the failure of two monotherapies, in particular if ETX has been used as a first line or second line monotherapy [41], it is usual to consider associating two or more antiepileptic drugs, as for other epileptic syndromes [42].

An increased efficacy has been demonstrated when ETX and VPA are combined because VPA can decrease the clearance of ETX [43]. The association of VPA and LTG must be titrated cautiously although its synergic mechanism can be useful in controlling monotherapy resistant forms of CAE [44]. Moreover, the indication to genetic analysis in patients with absences is controversial whereas in those cases of CAE that are refractory to first line therapy, some authors suggest to carry out a genetic screening in particular to detect Glucose transporter type 1 deficiency syndrome (Glut1D) [45,46]. In particular, Glut1D should be suspected and tested for when absence seizures are associated with irregular ictal EEG discharges and other features such as drug resistance and worsening during fasting as epileptic phenotypes mimicking absences are more frequently reported in familial cases of Glut1D [46]. On the other hand, an additional characteristic of absence seizures is that they are assumed to be almost exclusively hereditary and understanding how each genetic mutation converges on a similar phenotype helps illuminate the disease process and could ultimately identify potential strategies for treatment, in particular for refractory cases [47].

### 3.3. New Frontiers in Therapy-Resistant CAE Forms

Among older antiepileptic drugs, clobazam, clonazepam and acetazolamide have been traditionally used as second-line therapy for CAE or as adjunctive agents [48,49]. Over the past years, several new antiepileptic drugs such as levetiracetam, topiramate and zonisamide have become available for the treatment of resistant CAE, exhibiting a good efficacy and tolerability in clinical trials [50]. However, trials comparing the effects of these newer drugs to traditional therapy such as ETX and VPA are still missing (Table 1).

A small Italian multicenter, prospective, long-term, open-label treatment study published in 2008 enrolled 21 patients with typical absence seizures to undergo treatment with Levetiracetam (LEV) in monotherapy [26]. The basis of this study was that levetiracetam is currently used as monotherapy or as an adjunctive therapy in the treatment of various generalized epilepsies. LEV binds to a specific membrane binding site in the brain and does not affect glutamate or GABA-mediated synaptic transmission, nor modulation of voltage-dependent sodium or T-type calcium currents [51,52]. It’s effect is related to a-amino-3- hydroxy-5-methyl-4-isoxazole propionic acid receptor channels in mouse cortical neurons in culture [53]. The results of this study, although larger double blind studies are still awaited in order to confirm these findings, suggest that monotherapy with levetiracetam could be well tolerated and effective in patients with childhood absence epilepsy and juvenile absence epilepsy [26]. Moreover, a larger double blind, randomized, controlled clinical trial was conducted to compare the efficacy of levetiracetam to placebo in newly diagnosed CAE suggesting that levetiracetam provides a modest efficacy against absence seizures in the case of CAE [54]. However, the use of Levetiracetam in absence seizures still remains controversial and some Authors reported an aggravation of absence seizures when prescribing this therapy [55].

Topiramate (TPM) acts through ion-channel blockade as well as enhancing GABA-ergic inhibitory synaptic transmission and inhibiting excitatory pathways [1]. An open-label, pilot study conducted in 2002 evaluated the use of topiramate (at the dosage of 5 mg/kg/day) in five children with CAE and suggested that TPM may be useful for the treatment of childhood absence epilepsy [24]. Previously in 1999 TPM was effective for the treatment of 48% of enrolled patients with absence seizures in a randomized, placebo-controlled study [56]. However, another more recent pilot study conducted in 2011 that enrolled 12 patients with CAE was prematurely discontinued due to the inefficacy of topiramate monotherapy for the treatment of CAE [57].

Zonisamide (ZSN) acts by inhibiting carbonic anhydrase and by blocking T-type calcium channels as well as voltage-sensitive sodium channels [58]. Data regarding the use of this antiepileptic drug are still extremely scarce but a chart review that investigated the efficacy of zonisamide in 45 pediatric patients with CAE reported a 51.1% achievement of freedom from seizures, suggesting the possible use of this drug for the treatment of CAE [25].

The pharmaco-resistance of up to 30% of treated children with CAE and the detection of neuropsychiatric conditions on follow up, along with the adverse effects reported for many of the current antiepileptic drugs, require scientific research to focus on novel treatments [59]. Recent studies have underlined that adjunctive Perampanel can be useful in the treatment of absence seizures in the context of idiopathic generalized epilepsies [60] but extensive data are still missing. Concerning new frontier therapies there is a new drug (EpidiolexVR), an oral form of phytocannabinoid that is currently in phase 2 trials in children with pharmaco-resistant absence seizures; however there are no publicly reported data on the use of this drug for absence animal models and its possible neuropsychological effects in children and adolescents remains unclear [59,61]. Concerning non-pharmacological treatment, experimental animal models have identified highly excitable cortical zones that could be a future therapeutic target for techniques such as radiosurgery, ablation techniques or high frequency electrical subcortical or cortical stimulation [62]. Vagus nerve stimulation and ketogenic diet can also be considered in the management of pharmacological resistant forms of CAE. In particular, a ketogenic diet has been reported as successful in two scientific papers [63,64] although data are still extremely limited. Ketogenic diet is a medically supervised high-fat, low carbohydrate and moderate-protein diet that is used in many refractory epileptic syndromes [65]. A correct management of the ketogenic diet in pediatric patients with drug resistant epilepsy is important from the beginning to avoid side effects [66]. Thus, the initiation and maintenance of the treatment are the result of concomitant efforts of pediatric neurologists, dieticians, families and other caregivers. Moreover, it has also been suggested that different kinds of e-health applications should be used simultaneously, as complementary resources, to improve epileptic patient outcomes in the management of the ketogenic diet [67]. On the other hand, concerning vagus nerve stimulation, to our knowledge only Arya et al. [68] reported success with this technique on a very small number of patients with drug-resistant CAE.

### 3.4. Long Term Prognosis

The clinical course of children with CAE varies and basically includes three possible scenarios: terminal remission ≥ 12 months (occurring in about 65–82% of patients), persistent refractory CAE (occurring in about 11% of patients) and a change in epilepsy syndrome (about 15% of patients) [70]. There are very few long-term population-based studies in literature addressing the prognosis of CAE. Nevertheless, CAE generally has a high rate of long-term seizure freedom compared to other childhood onset epilepsies, occurring principally between 10 and 14 years of age. Concerning treatment effectiveness, Berg et al. [28] performed a prospective study evaluating the long-term prognosis of CAE considering the initial treatment received. In particular, in this study success rates were identical in the patient group treated with ETX to that treated with VPA but with the passing years, the number of patients treated with ETX and in complete remission was significantly higher than those in complete remission treated with VPA [28]. Moreover, a statistically significant correlation has been observed between cognitive impairment, longer duration of disease and higher frequency of seizures [69]. The most common neuropsychiatric comorbidities of CAE are subtle cognitive or language impairments, attention deficit hyperactivity disorder (ADHD) and anxiety [35,71]. It is therefore highly recommendable to consider screening for behavioral and psychological issues during the choice of treatment and during long term follow-up of CAE patients.

## 4. Conclusions

CAE is a common and easily diagnosable pediatric epileptic syndrome whose initial treatment of choice remains ethosuximide, followed by valproic acid in the case of treatment failure. There are few studies concerning the pharmacological-resistant forms and the possible neuropsychological implications of affected children.

Childhood absence epilepsy should not be considered so “benign” and future studies are certainly needed to address and prevent the later psychosocial comorbidities associated with this disease, along with the not so rare eventuality of a possible resistance to traditional therapy.

## Figures and Tables

**Table 1:** Possible therapeutic options for first line therapy-resistant CAE forms.

**Older therapeutic options for resistant CAE**	**Efficacy and characteristics**
Clobazam, Clonazepam and Acetazolamide	Clonazepam most frequently used but benzodiazepines have invalidating side effects and possible development of tolerance [58]. Only used in association with first line CAE therapy. Acetazolamide rarely used due to important adverse effects (kidney stones) [58].
**New therapeutic options** **for resistant CAE**	**Efficacy and characteristics**
Levetiracetam	Contrasting data regarding efficacy; possibly used in monotherapy [26,55]. Can be associated with VPA,LTG, ETX [69]. Most promising drug for future studies [58].
Topiramate	Not efficacious in monotherapy [57]. Can be associated with VPA,LTG, ETX [69].
Zonisamide	Possibly used in monotherapy [25]; studies are necessary for possible associations with other antiepileptic drugs.
**Experimental therapeutic options** **for resistant CAE**	**Efficacy and characteristics**
Perampanel; EpidiolexVR	Perampanel as adjunctive therapy in idiopathic generalized epilepsy with absences, no evidence yet for treatment of CAE [60]. Epidiolex VR only in animal models [59].

## References

[1] Matricardi S., Verrotti A., Chiarelli F., Cerminara C., Curatolo P. (2014). Current advances in childhood absence epilepsy. Pediatr. Neurol..

[2] Verrotti A., D’Alonzo R., Rinaldi V.E., Casciato S., D’Aniello A., Di Gennaro G. (2017). Childhood absence epilepsy and benign epilepsy with centro-temporal spikes: A narrative review analysis. World J. Pediatr..

[3] Hughes J.R. (2009). Absence seizures: A review of recent reports with new concepts. Epilepsy Behav..

[4] Guilhoto L.M. (2017). Absence epilepsy: Continuum of clinical presentation and epigenetics?. Seizure.

[5] Garzon P., Lemelle L., Auvin S. (2016). Childhood absence epilepsy: An update. Arch. Pediatr. Organe Off. Soc. Francaise Pediatr..

[6] Verrotti A., Laino D., Rinaldi V.E., Suppiej A., Giordano L., Toldo I., Margari L., Parisi P., Rizzo R., Matricardi S. (2016). Clinical dissection of childhood occipital epilepsy of Gastaut and prognostic implication. Eur. J. Neurol..

[7] Caraballo R.H., Sologuestua A., Grañana N., Adi J.N., Cersósimo R.O., Mazza E., Foster O., Fejerman N. (2004). Idiopathic occipital and absence epilepsies appearing in the same children. Pediatr. Neurol..

[8] Giordano L., Tambucci R., Cocco I.E., Angriman M., Coppola G., Operto F.F., Farello G., Savasta S., Belcastro V., Verrotti A. (2020). Infantile spasms followed by childhood absence epilepsy: A case series. Seizure.

[9] Verrotti A., Casciato S., Spalice A., Carotenuto M., Striano P., Parisi P., Zamponi N., Savasta S., Rinaldi V.E., D’Alonzo R. (2017). Coexistence of childhood absence epilepsy and benign epilepsy with centrotemporal spikes: A case series. Eur. J. Paediatr. Neurol. EJPN Off. J. Eur. Paediatr. Neurol. Soc..

[10] Crunelli V., Leresche N. (2002). Childhood absence epilepsy: Genes, channels, neurons and networks. Nat. Rev. Neurosci..

[11] Yalçın O. (2012). Genes and molecular mechanisms involved in the epileptogenesis of idiopathic absence epilepsies. Seizure.

[12] Thakran S., Guin D., Singh P., Singh P., Kukal S., Rawat C., Yadav S., Kushwaha S.S., Srivastava A.K., Hasija Y. (2020). Genetic Landscape of Common Epilepsies: Advancing towards Precision in Treatment. Int. J. Mol. Sci..

[13] Glauser T.A., Holland K., O’Brien V.P., Keddache M., Martin L.J., Clark P.O., Cnaan A., Dlugos D., Hirtz D.G., Shinnar S. (2017). Pharmacogenetics of antiepileptic drug efficacy in childhood absence epilepsy. Ann. Neurol..

[14] Tenney J.R., Kadis D.S., Agler W., Rozhkov L., Altaye M., Xiang J., Vannest J., Glauser T.A. (2018). Ictal connectivity in childhood absence epilepsy: Associations with outcome. Epilepsia.

[15] Glauser T.A., Cnaan A., Shinnar S., Hirtz D.G., Dlugos D., Masur D., Clark P.O., Capparelli E.V., Adamson P.C. (2010). Childhood Absence Epilepsy Study Group Ethosuximide, valproic acid, and lamotrigine in childhood absence epilepsy. N. Engl. J. Med..

[16] Vining E.P.G., Thio L.L. (2013). Absence in childhood absence epilepsy: The horse is out of the barn. Neurology.

[17] Auvin S. (2016). Advancing pharmacologic treatment options for pharmacologic treatment options for children with epilepsy. Expert Opin. Pharmacother..

[18] Shinnar R.C., Shinnar S., Cnaan A., Clark P., Dlugos D., Hirtz D.G., Hu F., Liu C., Masur D., Weiss E.F. (2017). Pretreatment behavior and subsequent medication effects in childhood absence epilepsy. Neurology.

[19] Matricardi S., Deleo F., Ragona F., Rinaldi V.E., Pelliccia S., Coppola G., Verrotti A. (2016). Neuropsychological profiles and outcomes in children with new onset frontal lobe epilepsy. Epilepsy Behav..

[20] Stafstrom C.E., Sun L.R., Kossoff E.H., Dabrowski A.K., Singhi S., Kelley S.A. (2021). Diagnosing and managing childhood absence epilepsy by telemedicine. Epilepsy Behav..

[21] Arsov T., Mullen S.A., Damiano J.A., Lawrence K.M., Huh L.L., Nolan M., Young H., Thouin A., Dahl H.-H.M., Berkovic S.F. (2012). Early onset absence epilepsy: 1 in 10 cases is caused by GLUT1 deficiency. Epilepsia.

[22] Wheless J.W., Clarke D.F., Arzimanoglou A., Carpenter D. (2007). Treatment of pediatric epilepsy: European expert opinion, 2007. Epileptic Disord. Int. Epilepsy J. Videotape.

[23] Kessler S.K., McGinnis E. (2019). A Practical Guide to Treatment of Childhood Absence Epilepsy. Paediatr. Drugs.

[24] Cross J.H. (2002). Topiramate monotherapy for childhood absence seizures: An open label pilot study. Seizure.

[25] Wilfong A., Schultz R. (2005). Zonisamide for absence seizures. Epilepsy Res..

[26] Verrotti A., Cerminara C., Domizio S., Mohn A., Franzoni E., Coppola G., Zamponi N., Parisi P., Iannetti P., Curatolo P. (2008). Levetiracetam in absence epilepsy. Dev. Med. Child Neurol..

[27] Kanner A.M., Ashman E., Gloss D., Harden C., Bourgeois B., Bautista J.F., Abou-Khalil B., Burakgazi-Dalkilic E., Llanas Park E., Stern J. (2018). Practice guideline update summary: Efficacy and tolerability of the new antiepileptic drugs I: Treatment of new-onset epilepsy: Report of the Guideline Development, Dissemination, and Implementation Subcommittee of the American Academy of Neurology and the American Epilepsy Society. Neurology.

[28] Berg A.T., Levy S.R., Testa F.M., Blumenfeld H. (2014). Long-term seizure remission in childhood absence epilepsy: Might initial treatment matter?. Epilepsia.

[29] Brigo F., Igwe S.C. (2017). Ethosuximide, sodium valproate or lamotrigine for absence seizures in children and adolescents. Cochrane Database Syst. Rev..

[30] Posner E.B., Mohamed K., Marson A.G. (2003). Ethosuximide, sodium valproate or lamotrigine for absence seizures in children and adolescents. Cochrane Database Syst. Rev..

[31] Coulter D.A., Huguenard J.R., Prince D.A. (1989). Characterization of ethosuximide reduction of low-threshold calcium current in thalamic neurons. Ann. Neurol..

[32] Smith G.A., McKauge L., Dubetz D., Tyrer J.H., Eadie M.J. (1979). Factors influencing plasma concentrations of ethosuximide. Clin. Pharmacokinet..

[33] Vining E.P.G. (2010). Ethosuximide in childhood absence epilepsy--older and better. N. Engl. J. Med..

[34] Romoli M., Mazzocchetti P., D’Alonzo R., Siliquini S., Rinaldi V.E., Verrotti A., Calabresi P., Costa C. (2019). Valproic Acid and Epilepsy: From Molecular Mechanisms to Clinical Evidences. Curr. Neuropharmacol..

[35] Verrotti A., Matricardi S., Rinaldi V.E., Prezioso G., Coppola G. (2015). Neuropsychological impairment in childhood absence epilepsy: Review of the literature. J. Neurol. Sci..

[36] Zaccara G., Franciotta D., Perucca E. (2007). Idiosyncratic adverse reactions to antiepileptic drugs. Epilepsia.

[37] Leach M.J., Marden C.M., Miller A.A. (1986). Pharmacological studies on lamotrigine, a novel potential antiepileptic drug: II. Neurochemical studies on the mechanism of action. Epilepsia.

[38] Frank L.M., Enlow T., Holmes G.L., Manasco P., Concannon S., Chen C., Womble G., Casale E.J. (1999). Lamictal (lamotrigine) monotherapy for typical absence seizures in children. Epilepsia.

[39] Holmes G.L., Frank L.M., Sheth R.D., Philbrook B., Wooten J.D., Vuong A., Kerls S., Hammer A.E., Messenheimer J. (2008). Lamotrigine monotherapy for newly diagnosed typical absence seizures in children. Epilepsy Res..

[40] Schlumberger E., Chavez F., Palacios L., Rey E., Pajot N., Dulac O. (1994). Lamotrigine in treatment of 120 children with epilepsy. Epilepsia.

[41] Cnaan A., Shinnar S., Arya R., Adamson P.C., Clark P.O., Dlugos D., Hirtz D.G., Masur D., Glauser T.A. (2017). Childhood Absence Epilepsy Study Group Second monotherapy in childhood absence epilepsy. Neurology.

[42] Auvin S. (2011). Management of childhood epilepsy. Presse Med. Paris Fr. 1983.

[43] Pisani F., Narbone M.C., Trunfio C., Fazio A., La Rosa G., Oteri G., Di Perri R. (1984). Valproic acid-ethosuximide interaction: A pharmacokinetic study. Epilepsia.

[44] Brodie M.J., Yuen A.W. (1997). Lamotrigine substitution study: Evidence for synergism with sodium valproate? 105 Study Group. Epilepsy Res..

[45] Mullen S.A., Suls A., De Jonghe P., Berkovic S.F., Scheffer I.E. (2010). Absence epilepsies with widely variable onset are a key feature of familial GLUT1 deficiency. Neurology.

[46] Ragona F., Matricardi S., Castellotti B., Patrini M., Freri E., Binelli S., Granata T. (2014). Refractory absence epilepsy and glut1 deficiency syndrome: A new case report and literature review. Neuropediatrics.

[47] Maheshwari A., Noebels J.L. (2014). Monogenic models of absence epilepsy: Windows into the complex balance between inhibition and excitation in thalamocortical microcircuits. Prog. Brain Res..

[48] Panayiotopoulos C.P. (2001). Treatment of typical absence seizures and related epileptic syndromes. Paediatr. Drugs.

[49] Hitiris N., Brodie M.J. (2005). Evidence-based treatment of idiopathic generalized epilepsies with older antiepileptic drugs. Epilepsia.

[50] Striano P., Minetti C. (2010). Epilepsy: Old drugs do the trick in childhood absence epilepsy. Nat. Rev. Neurol..

[51] Noyer M., Gillard M., Matagne A., Hénichart J.P., Wülfert E. (1995). The novel antiepileptic drug levetiracetam (ucb L059) appears to act via a specific binding site in CNS membranes. Eur. J. Pharmacol..

[52] Rigo J.-M., Hans G., Nguyen L., Rocher V., Belachew S., Malgrange B., Leprince P., Moonen G., Selak I., Matagne A. (2002). The anti-epileptic drug levetiracetam reverses the inhibition by negative allosteric modulators of neuronal GABA- and glycine-gated currents. Br. J. Pharmacol..

[53] Carunchio I., Pieri M., Ciotti M.T., Albo F., Zona C. (2007). Modulation of AMPA receptors in cultured cortical neurons induced by the antiepileptic drug levetiracetam. Epilepsia.

[54] Fattore C., Boniver C., Capovilla G., Cerminara C., Citterio A., Coppola G., Costa P., Darra F., Vecchi M., Perucca E. (2011). A multicenter, randomized, placebo-controlled trial of levetiracetam in children and adolescents with newly diagnosed absence epilepsy. Epilepsia.

[55] Auvin S., Chhun S., Berquin P., Ponchel E., Delanoë C., Chiron C. (2011). Aggravation of absence seizure related to levetiracetam. Eur. J. Paediatr. Neurol. EJPN Off. J. Eur. Paediatr. Neurol. Soc..

[56] Biton V., Montouris G.D., Ritter F., Riviello J.J., Reife R., Lim P., Pledger G. (1999). A randomized, placebo-controlled study of topiramate in primary generalized tonic-clonic seizures. Topiramate YTC Study Group. Neurology.

[57] Piña-Garza J.E., Schwarzman L., Wiegand F., Hulihan J. (2011). A pilot study of topiramate in childhood absence epilepsy. Acta Neurol. Scand..

[58] Posner E. (2006). Pharmacological treatment of childhood absence epilepsy. Expert Rev. Neurother..

[59] Crunelli V., Lőrincz M.L., McCafferty C., Lambert R.C., Leresche N., Di Giovanni G., David F. (2020). Clinical and experimental insight into pathophysiology, comorbidity and therapy of absence seizures. Brain J. Neurol..

[60] Trinka E., Lattanzi S., Carpenter K., Corradetti T., Nucera B., Rinaldi F., Shankar R., Brigo F. (2021). Exploring the Evidence for Broad-Spectrum Effectiveness of Perampanel: A Systematic Review of Clinical Data in Generalised Seizures. CNS Drugs.

[61] Stockings E., Zagic D., Campbell G., Weier M., Hall W.D., Nielsen S., Herkes G.K., Farrell M., Degenhardt L. (2018). Evidence for cannabis and cannabinoids for epilepsy: A systematic review of controlled and observational evidence. J. Neurol. Neurosurg. Psychiatry.

[62] Luijtelaar G., van Zobeiri M., Lüttjohann A., Depaulis A. (2017). Experimental Treatment Options in Absence Epilepsy. Curr. Pharm. Des..

[63] Groomes L.B., Pyzik P.L., Turner Z., Dorward J.L., Goode V.H., Kossoff E.H. (2011). Do patients with absence epilepsy respond to ketogenic diets?. J. Child Neurol..

[64] Clemens Z., Kelemen A., Fogarasi A., Tóth C. (2013). Childhood absence epilepsy successfully treated with the paleolithic ketogenic diet. Neurol. Ther..

[65] Thammongkol S., Vears D.F., Bicknell-Royle J., Nation J., Draffin K., Stewart K.G., Scheffer I.E., Mackay M.T. (2012). Efficacy of the ketogenic diet: Which epilepsies respond?. Epilepsia.

[66] Marchiò M., Roli L., Lucchi C., Costa A.M., Borghi M., Iughetti L., Trenti T., Guerra A., Biagini G. (2019). Ghrelin Plasma Levels After 1 Year of Ketogenic Diet in Children With Refractory Epilepsy. Front. Nutr..

[67] Costa A.-M., Marchiò M., Bruni G., Bernabei S.M., Cavalieri S., Bondi M., Biagini G. (2021). Evaluation of E-Health Applications for Paediatric Patients with Refractory Epilepsy and Maintained on Ketogenic Diet. Nutrients.

[68] Arya R., Greiner H.M., Lewis A., Mangano F.T., Gonsalves C., Holland K.D., Glauser T.A. (2013). Vagus nerve stimulation for medically refractory absence epilepsy. Seizure.

[69] Franzoni E., Matricardi S., Di Pisa V., Capovilla G., Romeo A., Tozzi E., Pruna D., Salerno G.G., Zamponi N., Accorsi P. (2015). Refractory absence seizures: An Italian multicenter retrospective study. Eur. J. Paediatr. Neurol..

[70] Nabbout R., Andrade D.M., Bahi-Buisson N., Cross H., Desquerre I., Dulac O., Granata T., Hirsch E., Navarro V., Ouss L. (2017). Outcome of childhood-onset epilepsy from adolescence to adulthood: Transition issues. Epilepsy Behav..

[71] Caplan R., Siddarth P., Stahl L., Lanphier E., Vona P., Gurbani S., Koh S., Sankar R., Shields W.D. (2008). Childhood absence epilepsy: Behavioral, cognitive, and linguistic comorbidities. Epilepsia.

